# Scrub Typhus in Himalayas

**DOI:** 10.3201/eid1210.051697

**Published:** 2006-10

**Authors:** Sanjay K. Mahajan, Jean-Marc Rolain, Rajesh Kashyap, Diprabhanu Bakshi, Vijay Sharma, Bhupal Singh Prasher, Lal Singh Pal, Didier Raoult

**Affiliations:** * IG Medical College, Shimla, Himachal Pradesh, India;; † Université de la Méditerranée, Marseille, France;; ‡ Defence Research and Development Establishment, Gwalior, Madhya Pradesh, India

**Keywords:** Rickettsia, Orientia tsutsugamushi, India, Himachal Pradesh, Himalaya, dispatch

## Abstract

Himachal Pradesh state of India is situated in the outer Himalayan ranges. During the rainy season, several cases of acute febrile illness of unknown origin occurred. Orientia tsutsugamushi was identified as the causative agent by microimmunofluorescence and PCR. Two new genotypes of O. tsutsugamushi were identified in the region.

Microimmunofluorescence (MIF) assay is the test of choice to diagnose rickettsial diseases (*1**,**2*), but more sensitive serologic tests and real-time quantitative PCR are expected to increase the number of cases diagnosed (*3*). Rickettsial diseases have been reported from various regions of India in the recent past (*4**–**6*); in the past few years, acute febrile illness with multiple organ involvement was diagnosed in several patients in our area. Results of tests for common causes of fever were negative, as were results of serologic tests for infectious mononucleosis, immunoglobulin M (IgM) for dengue fever, and IgM for leptospirosis. Sixteen of 31 serum samples from patients with suspected scrub typhus had titers 40–160 on Weil-Felix agglutination test with *Proteus* OXK antigen in 2003.

Himachal Pradesh is a mountainous state in northern India, situated in the outer Himalayas, with altitudes 350–7,000 m above sea level. It is the least urbanized state in India. During the rainy season, areas at lower altitudes have an average temperature of 20°C to 35°C, which is conducive to the spread of arthropod vectors. We recently reported an outbreak of scrub typhus in these areas (*7*). In an entomologic study in Himachal Pradesh, vector species *Leptotrombidium deliense* and *Gahrliepia* (*schoengastilla*) spp. were recorded (*8*). The aim of the present study was to retrospectively diagnose suspected rickettsial disease (scrub typhus) by using MIF assay and molecular methods in patients with acute febrile illness of unknown origin.

## The Study

The study was conducted from July through October 2004. Scrub typhus was suspected by clinical manifestations such as febrile illness or fever with rash or eschar. After giving informed consent, patients filled out questionnaires about potential chigger exposure and symptoms or signs consistent with scrub typhus. Blood samples were taken from all patients for total blood cell count, biochemical analysis, serologic diagnosis, and molecular assays. This study was exempt from human subject review.

When they sought treatment, 5 patients had been symptomatic for 5 to 7 days, 14 had been symptomatic for 8 to 14 days, and the remaining 2 had been symptomatic for 18 to 25 days. Blood samples were collected at the time of admission to the hospital, and no serial assays were performed. All patients with clinical features that suggested scrub typhus received antirickettsial drugs (doxycycline/azithromycin) empirically.

Two serologic tests were used to confirm infections. The Weil-Felix *Proteus* agglutination assay with *Proteus vulgaris* OX-19 and OX-2 and *P. mirabilis* OX-K strains (Wellcome Diagnostics, Dartford, UK) was performed on each sample; a titer >80 was considered positive. Serum specimens were stored at –20°C. Serum specimens were tested by MIF assay with a panel of 11 rickettsial antigens, including spotted fever group (SFG) rickettsiae (*Rickettsia japonica*, *R. helvetica*, *R. slovaca*, *R. conorii* subsp. *indica*, *R. honeï*, *R. heilongjangensis*, and *R. felis*), *R. typhi*, and *Orientia tsutsugamushi* (Gilliam, Karp, Kato and Kawasaki strains) (*3*). The MIF assay was considered positive if antibody titers were >128 for IgG and >64 for IgM or if seroconversion was demonstrated (*9**,**10*).

DNA was extracted from the blood sample (buffy coat) by using QIAamp DNA Mini Kit (Qiagen GmbH, Hilden, Germany) according to the manufacturer's instructions. Two amplification reactions were performed: 1) a real-time quantitative PCR with a TaqMan probe targeting the 47-kDa outer membrane protein with primers and probe previously described (*11*) and 2) a standard PCR targeting the 56-kDa protein with forward and reverse primers (OtsuF: 5´-AATTGCTAGTGCAATGTCTG-3´ and OtsuR: 5´-GGCATTATAGTAGGCTGAG-3´). The primers were purchased from Eurobio (Paris, France). The success of the amplification was confirmed by resolution of the products by electrophoresis on 1% agarose gel (Sigma Chemical Co., Saint Louis, MO, USA) in 1× Tris borate EDTA buffer for products of the 56-kDa gene. The sizes of the PCR-amplified products were determined by comparison with a molecular weight standard (Boehringer, Manheim, Germany) under UV light after ethidium bromide staining.

The PCR products were purified by using the QIAquick PCR Purification Kit (Qiagen) according to the manufacturer's instructions. Sequencing reactions were performed with a DNA sequencing kit, dRhodamine Terminator Cycle Sequencing Ready Reaction Mix (Applied Biosystems, Foster City, CA, USA). Sequencing was performed on an ABI PRISM 310 DNA Sequencer (Applied Biosystems). The sequences were identified by comparison with sequences available in GenBank by using the BLAST software (http://www.ncbi.nlm.nih.gov/BLAST/).

In the MIF assay, samples from 21 patients showed positive antibody titers (both IgG and IgM) to *O. tsutsugamushi* (Gilliam, Karp, Kato, and Kawasaki strains). Twenty-eight of 51 serum samples had titers 80–320 to *Proteus* OXK antigen on Weil-Felix test, of which only 13 had titers on MIF. Moreover, samples from 15 patients with titers on Weil-Felix did not show titers on MIF, and samples from 8 patients without titers on Weil-Felix showed titers on MIF. None of the patients had a positive serologic test result for SFG rickettsioses. Real-time quantitative PCR to *O. tsutsugamushi* was positive in anticoagulated blood of 3 patients, and this result was confirmed by using primers for the 56-kDa antigen gene of *O. tsutsugamushi*. Patient 1 had a sequence (GenBank accession no. DQ530440) that matched the Karp type, close to strain LA-1 isolated in 1993 in Malaysia from mites (*12*). The sequence obtained from patient 2 (GenBank accession no. DQ530441) matched the sequence between the JG type and a recently described new subtype called Saitama, described in Japan (*13*). The results of MIF assays are shown in Table 1. The clinical features and laboratory abnormalities found in these 21 patients are shown in Table 2.

**Table 1:** MIF assay results from 21 patients with suspected scrub typhus, Himalayas, 2004*

Patient no.	MIF titers (IgG/IgM)		Outcome
	Orientia Kato/Gilliam	Orientia Kawasaki	
1	128/0	512/0	Died†
2	128/0	256/0	Improved†
3	1,024/0	1,024/0	Improved
4	128/64	256/64	Improved
5	2,048/0	2,048/0	Improved
6	2,048/1024	2,048/1,024	Improved
7	1,024/128	512/128	Improved
8	2,048/64	512/0	Improved
9	512/0	512/0	Died†
10	2,048/0	2,048/0	Improved
11	1,024/256	1,024/256	Improved
12	256/128	512/256	Improved
13	2,048/0	2,048/0	Improved
14	0/128	0/128	Improved
15	512/256	256/256	Died
16	64/128	128/128	Improved
17	512/0	512/0	Improved
18	2,048/0	2,048/0	Improved
19	512/64	0/64	Improved
20	64/64	0/64	Improved
21	128/256	0/256	Improved

*MIF, microimmunofluorescence; Ig, immunoglobulin. IgM was considered positive at a titer of 64; IgG was considered positive at a titer of 128. †Positive PCR result.

**Table 2:** Distribution of clinical features in 21 patients with suspected scrub typhus, Himalayas, 2004

Clinical feature*	Men (n = 13)	Women (n = 8)	Total (%)
Fever	13	8	21 (100)
Chills and rigor	10	5	15 (71.4)
Vomiting	6	3	9 (42.8)
Myalgia	4	4	8 (38.0)
Headache	4	4	8 (38.0)
Altered sensorium	2	3	5 (23.8)
Lymphadenopathy	9	2	11 (52.3)
Jaundice	5	6	11 (52.3)
Hepatomegaly	6	3	9 (42.8)
Congested eyes	5	2	7 (33.3)
Splenomegaly	4	3	7 (33.3)
Abdominal pain	2	4	6 (28.5)
Seizures	2	2	4 (19.0)
Cough	2	2	4 (19.0)
Abnormal bleeding	1	2	3 (14.2)
Eschar	2	0	2 (9.5)
Meningeal signs	1	1	2 (9.5)
Rash	2	0	2 (9.5)
Elevated transaminase levels	7	7	14 (66.7)
Renal dysfunction	8	6	14 (66.7)
Proteinuria	5	3	8 (38.1)
CSF abnormalities†	1	2	3 (14.3)
Acute RDS	1	1	2 (9.5)

*CSF, cerebrospinal fluid; RDS, respiratory distress syndrome. †Elevated protein level and increased lymphocyte count.

By MIF assay, 11 of 21 samples had positive titers of both IgG and IgM; 3 were positive for IgG (but not IgM) and were positive on PCR. These 14 patients had acute infection and are considered to have had scrub typhus, whereas the remaining 7 patients with only IgG titers are considered as probable scrub typhus case-patients. In cases of primary infection with *O. tsutsugamushi*, IgM appears at the end of the first week, whereas IgG appears at the end of the second week. However, in the case of reinfection with *O. tsutsugamushi*, IgG is detectable by day 6, and IgM titers are variable. The absence of IgM in 10 samples can be attributable to previous antigenic conditioning from reinfection (*9*). Moreover all 3 patients with positive PCR results had IgG titers but not IgM titers, which suggests that some of the remaining 7 patients with only IgG might also have been acutely infected.

## Conclusions

We confirmed the diagnosis of scrub typhus in 21 patients from the Himalayas with several validated assays. PCR was performed in a few cases to further confirm *O. tsutsugamushi*, and we found that PCR was a good tool for molecular diagnosis, as recently reported (*6*). To the best of our knowledge, this is the first molecular detection of *O. tsutsugamushi* in southern Asia. The result was confirmed by using 2 different target genes in 2 different PCR assays. In our study, >2 different genotypes were identified; the phylogenetic position of 1 is between Karp and JP-1, and the other is between Saitama and JG type (Figure). Therefore, isolating these strains is recommended to increase understanding of the epidemiologic features of scrub typhus in India (*12*). In our study, an eschar, which is formed in few secondary infections (*2*), was noted in 9.5% of cases. Eschars are rare in Southeast Asian patients, and indigenous persons of typhus-endemic areas commonly have less severe illness, often without rash or eschar (*14*). The variation in cutaneous immunity has also been suggested as a possible explanation for the absence of an eschar in certain instances of scrub typhus (*15*).

**Figure:**
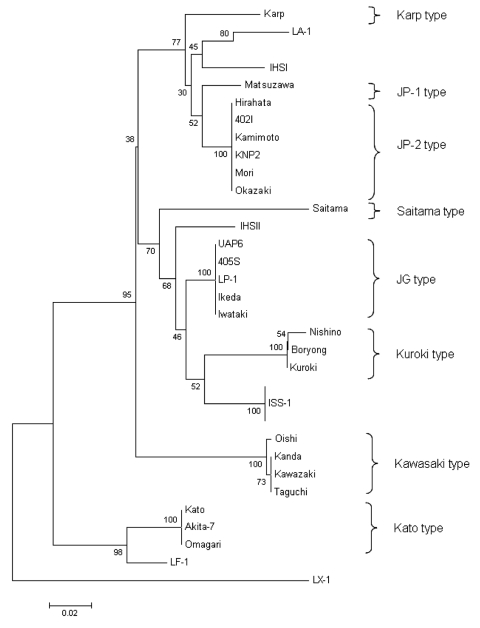
Phylogenetic tree produced by unweighted pair-group method with arithmetic means that shows the positions of IHS I and IHS II genotypes based on the partial 56-kDa sequence homologies. Numbers at nodes indicate bootstrap values, and the scale bar shows genetic distance of 0.02.

The literature mentions this disease in hilly regions of the Himalayas and the Shimla region in Himachal Pradesh (*5**,**7**,**15*), but specific data are not available. The disease must have been present in the area but was not noticed because, in the past, most cases of fever were treated with drugs like tetracycline and chloramphenicol, which effectively treat scrub typhus also. The incidence of pyrexia of unknown origin with multiple organ involvement has increased for the past few years, which prompted us to conduct this study. Increasing prevalence of scrub typhus has been reported from some Asian countries, which coincides with the widespread use of β-lactam antimicrobial drugs and urbanization in rural areas (*14*).
